# Reports on the Hospitals of the United Kingdom

**Published:** 1909-11-06

**Authors:** Henry Burdett


### REPORTS
### ON THE
### Hospitals of the United Kingdom.

BY SIR HENRY BURDETT, K.C.B., K.C.V.O.

### VII.—ROYAL DEVON AND EXETER HOSPITAL, EXETER.

THIS institution was founded in 1741, and a new wing was opened in 1897. It contains 200 beds, with an average occupancy of 155. Our first report on this institution was published in THE HOSPITAL of October 15, 1892. At that time, owing to much wear and tear, the appearance of the wards and offices was far behind that of a modern Poor Law infirmary, and the whole place needed immediately redecorating, renewing, and refurnishing. Two years later, in THE HOSPITAL of November 10, 1894, we published the report of a further inspection, and the public spirit exhibited by the local press and inhabitants of Exeter in the intervening period enabled us to report that the Devon and Exeter Hospital "has been so changed that its present condition compared with that of two years ago represents nothing less than a transformation scene, to the great credit of all concerned." Several matters, including the operation theatre unit, required to be dealt with, and the buildings were, of course, old, and many of them out of date. We had therefore special interest in inspecting this institution in September 1909. We found that the operation theatre unit has been remodelled, and that in making the changes one special feature of the original plan has been destroyed. This feature is an operation wing which contains a number of small wards, where patients after an operation can be retained so as to reduce the risk of removal from the theatre to a minimum. It is interesting to note in this connection that, owing to the ever increasing number of operations which are being performed in the voluntary hospitals of this country in the present day, and to the strain upon the surgeons and the resident medical and nursing staffs which this involves, it is highly probable that the old Exeter idea in regard to the operation building may again form a feature in future hospital construction, with the modification that wards with several beds in addition to single-bedded wards will be included. It is quite certain that as a matter of precaution, and on humanitarian and other grounds, it is essential that a patient who is removed from the theatre back to the ward after an operation must have the continuous attendance of one nurse at least until complete recovery has taken place from the anæsthetic. Where a large surgical ward is nursed by a sister, a charge nurse, and two probationers it must be manifestly difficult, if not impossible, with such a staff to attend completely to the requirements of operation cases when seven or more inmates of the same ward are operated upon in a day. Another point which is attracting attention at most hospitals at the present time is the inconvenience, to say the least, of having so many cases of operation returned to a ward amongst patients, several of whom may have to undergo an operation themselves. All these matters which have arisen out of the enormous growth in operative interference render it probable that material changes may necessarily take place in the plan of construction of hospitals in the future, such modifications reverting in a measure, at any rate, to the principle formerly represented by the operation wing at Exeter.

### WORK YET TO BE DONE.

In 1894 we earnestly insisted that the old buildings required further renovation and new sanitary fittings, changes which could best be brought about by a reconstruction of the then existing wards and the addition of a new wing for the reception of patients. This counsel was adopted by the authorities, and a new wing was added three years later in 1897. The wards of this wing were in marked contrast to the older portions of the building, and presented a bright and pleasant appearance, being well kept and in good order at the time of our visit. The reconstruction of the older wards has, however, not taken place. These are contained in a wing parallel with the new wing, and are old back-to-back wards, the atmosphere of which was most unsatisfactory from the want of effective ventilation and proper circulation of air at the time of our visit. Indeed, we were not surprised to hear that this vitiated atmosphere had a distinct effect upon the patients, and we are bound to say that the committee ought without hesitation or delay to close these wards and reconstruct this wing from end to end. The wards need opening out; the dividing wall should be removed as far as possible and arches substituted, whilst all the windows should be converted into louver windows with a central pivot action, so as to flush the wards continuously. The actual possibilities and best plan of reconstruction of these particular wards can be readily ascertained by a visit to the London Hospital at Whitechapel, where wards of a similar date and design have been transformed and their defects remedied so far as is structurally possible. It is not right to delay this relatively small reconstruction, because of its importance to the well-being and welfare of the patients, and to the reputation of the hospital and those responsible for its management. Anything we can do to help the committee to complete this most urgently needed reconstruction will be gladly done.

When these old wards have been reconstructed the committee should seriously take in hand the central block, which constitutes, we believe, the oldest portion of the hospital. Possibly the best course to take would be to pull the whole of it down and rebuild, so securing that every advantage may be taken of the light and air space available. If it is thought desirable to preserve the façade that might possibly be done, but we should hardly think local sentiment would attach much importance to this point. In any case, the condition of this portion of the building, and the urgent need which exists at the present time for better and proper office, sleeping, and other accommodation in a well-planned, modern, up-to-date administrative block, make it imperative that these old buildings should be taken in hand without a moment's avoidable delay.

### The Nursing Department and Home.

We are glad to note that the authorities have had the wisdom to maintain a large and efficient private staff, the services of which should be made available in the first instance for the supporters of the hospital and their families, a plan which we recommend, as it might considerably increase the revenue of the institution. The staff of nurses is large, there being at the time of our visit thirty-eight members of the private staff and forty-two working in the hospital itself. The accommodation for the nurses is, however, anything but creditable to the management. Why should the Devon and Exeter Hospital, which is one of the most celebrated, and was for many decades one of the largest and most important of English county hospitals, suffer its good name to be imperilled by ignoring the claims of the nursing staff, which every other modern hospital has recognised and provided for? There is such a lack of accommodation for the nurses in the home that they have to sleep practically all over the hospital. This scattering of the dormitories is not conducive to comfort, health, discipline, or, indeed, to anything which is desirable. Having regard to the size of the private nursing staff and its importance, and to the important medical and surgical work now done in the hospital itself, it is essential, if the reputation of this hospital is to be maintained, that an adequate nursing home should be provided at once. We hope that in deciding upon the plan of such a home infinite care will be taken to see that the practical details are thoroughly mastered and attended to, for it is very easy, as many existing homes prove, to waste a great deal of money upon such a building and to find that the accommodation provided is often inadequate when the bill is paid. Seeing that the provision of such a home has been delayed too long, the committee have an opportunity of showing their wisdom by providing that every development and improvement in the construction and arrangement of a nursing home shall be included in the plan of the new buildings at Exeter.

We were taken over the hospital by Miss Dixon, the assistant matron, who showed a thorough mastery of the details of the work, and evidently takes a keen interest in securing the best management and the utmost efficiency in all departments. She was, we believe, trained at Exeter, a fact which should appeal to the imagination of the committee, and make them resolve to do justice to their nursing staff, and to get them properly housed without further loss of time.

### A Remarkable Incident.

When we were inspecting the operation theatre, which is unusually large, we became acquainted with the fact that the probationers are arranged in rows in the theatre on the days when operations take place. We cannot say who is responsible for this extraordinary regulation, which seems to be in force, but we may say frankly that we regard it as a most improper and useless one, and are glad to be able to state that it has no existence in any other hospital with which we are acquainted. If the object of ranging probationers in rows in an operation theatre be to teach them the duties in regard to operation cases, every practical person must know that no such result can follow a practice which is unhygienic and undesirable for every reason. We hope, therefore, to hear that other and better methods of instructing probationers in the work appertaining to a nurse in regard to operation cases will be followed at Exeter. It would be interesting to know how such a practice has grown up, and still more interesting to know why the superintendent of nurses and the matron have allowed it to continue for so long.

### Finance.

The financial position, as shown by the accounts and report of this hospital, is not satisfactory. The main sources of income, *i.e.* subscriptions, congregational collections, and contributions from workpeople on Hospital Saturday, are practically stationary, whilst the donations fell off in 1908 compared with 1907 by about 30 per cent. There was a deficiency in 1907 of £3,988, and in 1908 of £1,948, as shown in the receipts and payments account for 1908. The diminished deficiency in the latter year is due to the crediting of all the legacies and special donations, amounting to upwards of £4,500, to extraordinary income and spending them. To state this is to show the serious financial position of the hospital at the present moment. We should like to make the financial condition even more apparent, but it is impossible to deal with the income and expenditure over a series of years, for the reason that the report contains no tables giving figures, and that it does not even state the average number of days each patient was resident, or the cost of each in- and out-patient. These are serious omissions, and should be rectified in future reports. The financial difficulties are due in no small measure, we imagine, to the scale on which in- and out-patient tickets are issued. One way out of the financial difficulty is undoubtedly to do what other hospitals have done in similar circumstances, namely, to revise the privileges of the governors. At the present time a subscriber of one guinea is entitled under the rules to recommend one in-patient each year. Now the average cost per in-patient at this hospital is £5 15s., and that cost would no doubt have been much higher, if this hospital did not retain its patients so long in the wards that the cost of their maintenance must average out at an unusually low figure. Thus, the average number of days each in-patient was resident in 1907, the latest figures we have, was 41 days, whereas an average of 21 days, or even less, should suffice, if the cases admitted are serious hospital cases and not mere chronics. This fact illustrates the danger of having too many beds in a hospital of this type. It is not, for instance, a good thing to have so many beds available that there is room to admit any case, however chronic, which can benefit a little by having good food and care in a hospital ward. The best administered hospitals aim at curing the greatest number of patients with the fewest number of beds in the shortest possible time. At Exeter to-day it would appear as if this process were reversed, for, instead of passing from 15 to 19 patients through each occupied bed each year, they only pass nine patients through each such bed. From this fact we might infer with some justice that the number of beds might be considerably reduced if the admissions were regulated by the rules which govern most modern hospitals of the present day.

### NEITHER BUSINESS NOR PHILANTHROPY.

But to revert to the tickets issued to subscribers and the cost of in-patients. The hospital at present needs an increased revenue of £3,000 a year. Where is that revenue to come from? We should say that the larger portion of it ought to be forthcoming, provided the committee take the governors into their confidence and point out to them quite plainly that the present scale of privileges is neither business nor philanthropy. What business is there in taking a guinea from an individual subscriber and handing him back £5 15s. in value by way of treatment? What philanthropy is there on the part of the governor who gives a guinea for the privilege of taking in return from the hospital funds £5 15s. per annum? Let us put these figures together and express the result in annual loss to the hospital under the present scale of governors' privileges. One thousand five hundred and nine in-patients were admitted to treatment in 1908. We have nowhere any information as to the average stay of the in-patients in 1908, or as to how many, if any, in-patients were admitted without ticket. On these points the report is silent, a fact which emphasises strongly the necessity for appointing a committee to obtain the reports of other large hospitals with a view to recast the present annual report and to make it include much valuable information now absent from its pages. Assuming that two-thirds, or, say, 1,000 patients, were admitted by ticket, that represents a cost to the hospital of £5,750. Towards the cost thus incurred of treating the patients sent in by subscribers, the subscribers contributed £1,050, representing a dead loss in a single year to the institution of £4,700. It is small wonder, in face of such a fact as this, that the income of the hospital is altogether inadequate to meet its expenditure. The wonder is that the present state of affairs has endured so long. We should recommend the governors to take this matter in hand, to appoint a small committee to take expert advice and to prepare a scheme revising the present scale of privileges which will place it upon a basis which will have at any rate a measure of business and philanthropy to rest upon. In this way the whole financial position might be placed upon a sound and permanent basis.

**The Salisbury Infirmary** and **Some Cottage Hospitals** will be reported on next week.

